# Clinical Effectiveness and Cost-Effectiveness of Blended Treatment for Major Depression Compared With Treatment as Usual Within Routine Care in Europe: A Noninferiority Randomized Controlled Trial

**DOI:** 10.2196/72866

**Published:** 2026-09-24

**Authors:** Heleen Riper, Annet Kleiboer, Adriaan Hoogendoorn, Judith Bosmans, Mieke Schulte, Kim Mathiasen, Jean Baptiste Hazo, Jerome Holtzmann, Karine Chevreul, David Daniel Ebert, Ingrid Titzler, Mathias Berking, Burkhardt Funk, Anneke van Schaik, Lise Kemmeren, Roman Cieslak, Ewelina Smoktunowicz, Anna Maj, Cristina Botella, Azucena Garcia-Palacios, Rosa Banos, Rocío Herrero, Gerhard Andersson, Naira Topooco, Kristofer Vernmark, Thomas Berger, Tobias Krieger, Arlinda Cerga-Pashoja, Asmae Doukani, Ricardo Araya, Eirini Karyotaki, Spyros Kolovos, Mark Hoogendoorn, Ward van Breda, Pepijn van de Ven, Artur Rocha, Gonçalo Gonçalves, Pim Cuijpers

**Affiliations:** 1 Department of Clinical, Neuro- and Developmental Psychology, Amsterdam Public Health Research Institute, Vrije Universiteit Amsterdam, Van der Boechorststraat 7, Amsterdam, 1081 BT, The Netherlands; 2 Amsterdam UMC, Location Vrije Universiteit Amsterdam, Department of Psychiatry, Amsterdam Public Health Research Institute, Amsterdam, The Netherlands; 3 Department of Health Sciences, Faculty of Earth and Life Sciences, Amsterdam Public Health Research Institute, Vrije Universiteit, Amsterdam, The Netherlands; 4 Department of Psychology, Education and Child Studies, Clinical Psychology, Erasmus School of Social and Behavioral Sciences, Erasmus University Rotterdam, Rotterdam, The Netherlands; 5 Department of Psychology and Behavioral Sciences, Aarhus University, Aarhus, Denmark; 6 ECEVE, UMR 1123, Inserm, Université Paris Cité, Paris, France; 7 Health Economics Research Unit, Inserm, University of Paris, Paris, France; 8 Service de Psychiatrie de l’Adulte, Centre Expert Dépression Résistante FondaMental, CHU Grenoble Alpes, Hôpital Nord, Grenoble, France; 9 Fondation FondaMental (FondaMental Foundation), Créteil, France; 10 Psychology and Digital Mental Health Care, TUM School of Medicine and Health, Technical University of Munich, Munich, Germany; 11 Department of Clinical Psychology and Psychotherapy, Friedrich-Alexander-Universität Erlangen-Nürnberg, Erlangen, Germany; 12 Institute of Psychology, UMIT TIROL, Private University for Health Sciences and Technology, Hall in Tirol, Austria; 13 Institute of Information Systems, Leuphana University, Lüneburg, Germany; 14 Department of Research and Innovation, GGZ InGeest Specialized Mental Health Care, Amsterdam, The Netherlands; 15 Faculty of Psychology, SWPS University, Warsaw, Poland; 16 Lyda Hill Institute for Human Resilience, University of Colorado Colorado Springs, Colorado Springs, CO, United States; 17 StresLab Research Centre, Institute of Psychology, SWPS University, Warsaw, Poland; 18 Department of Basic Psychology, Clinical and Psychobiology, Universitat Jaume I, Castellón de la Plana, Spain; 19 CIBERobn Physiopathology of Obesity and Nutrition, Instituto de Salud Carlos III, Madrid, Spain; 20 Department of Personality, Evaluation and Psychological Treatments, Universidad de Valencia, Valencia, Spain; 21 Department of Behavioral Sciences and Learning, Linköping University, Linköping, Sweden; 22 Lusófona University, HEI-Lab: Digital Human-EnvironmentInteraction Labs, Lisboa, Portugal; 23 Department of Health, Education and Technology, Luleå University of Technology, Luleå, Sweden; 24 Department of Psychology, Uppsala University, Uppsala, Sweden; 25 Department of Clinical Psychology and Psychotherapy, University of Bern, Bern, Switzerland; 26 Department of Population Health, London School of Hygiene & Tropical Medicine, London, United Kingdom; 27 School of Allied Health and Life Sciences, Faculty of Sport, Technology and Health Sciences, St Mary's University, Twickenham, United Kingdom; 28 Department of Health Service and Population Research, Institute of Psychiatry, Psychology & Neuroscience, King’s College London, London, United Kingdom; 29 Department of Clinical Psychology, University of Amsterdam, Amsterdam, The Netherlands; 30 Department of Computer Science, Vrije Universiteit Amsterdam, Amsterdam, The Netherlands; 31 Health Research Institute, University of Limerick, Limerick, Ireland; 32 Human-Centered Computing and Information Science, Institute for Systems Engineeringand Computers Technology and Science (INESCTEC), Porto, Portugal

**Keywords:** major depression disorder, cognitive behavioral therapy, blended treatment, cost-effectiveness, noninferiority randomized controlled trial, digital mental health

## Abstract

**Background:**

Cognitive behavioral therapy (CBT) is an effective and widely used treatment for major depressive disorder (MDD). However, access is limited by long waiting lists and a shortage of trained therapists. Evidence-based guided and self-guided digital interventions can address these challenges, but their large-scale adoption in routine primary and specialized mental health care has been slow. Blended CBT (bCBT), combining face-to-face therapy with structured, guided digital treatment modules, may increase treatment capacity while maintaining the benefits of therapist support.

**Objective:**

The European Comparative Effectiveness Research on Internet-based Depression Treatment study, conducted across 9 European countries, is the first large-scale comparative bCBT study for MDD. The hypothesis was that bCBT is clinically noninferior and cost-effective when compared with treatment as usual (TAU), which mainly consisted of face-to-face CBT.

**Methods:**

A multisite randomized controlled trial was conducted with a noninferiority margin of *d*=0.20. Main inclusion criteria were age ≥18 years, a diagnosis of MDD based on the Mini International Neuropsychiatric Interview, and a baseline Patient Health Questionnaire-9 (PHQ-9) score of ≥5. The primary outcome was the PHQ-9, with secondary outcomes including MDD remission at 12 months, therapeutic alliance, and costs. Intention-to-treat analyses were performed, and linear mixed modeling was used to assess intervention effects. Cost-effectiveness analyses were conducted from a health care perspective.

**Results:**

A total of 835 patients were included in the study. bCBT was shown to be noninferior to TAU on the primary PHQ-9 outcome during treatment (3 months after the baseline assessment, *d*=−0.26, 95% CI −0.43 to −0.09), at 6 months (posttreatment, *d*=−0.21, 95% CI −0.39 to−0.04), and at 12-month follow-up (*d*=−0.01, 95% CI −0.19 to 0.17). Furthermore, the bCBT group had a significantly lower likelihood of experiencing an MDD episode at 12 months (odds ratio 0.67, 95% CI 0.45‐0.99). Subgroup analyses indicated that participants with and with no antidepressant use at baseline in both groups benefited equally from their treatment. Deterioration rates (Reliable Change Index) were below 5% in both groups. bCBT appeared acceptable for patients and therapists, with a strong working alliance in both groups. From a health care perspective, bCBT was not cost-effective at 12 months' assessment, as its total costs were not significantly lower than TAU. However, the probability that bCBT is cost-effective is high (0.95) at a willingness to pay (WTP) of €3800 per improvement in the PHQ-9 score, and 0.76 at a WTP of €10,000 per MDD case prevented (average 2017 exchange rate was €1=US $1.13).

**Conclusions:**

bCBT offers an effective and safe digitally supported alternative to face-to-face TAU for patients with MDD and their therapists in routine clinical care. From a health care perspective, its cost-effectiveness depends on policymakers’ WTP for the additional clinical benefits achieved.

## Introduction

Globally, major depressive disorder (MDD) is among the most prevalent mental disorders, affecting approximately 4% of the population each year, corresponding to an estimated 332 million people in 2021 [1]. MDD is a leading cause of disability worldwide and imposes a substantial societal burden through increased health care usage and major productivity losses [2,3]. MDD is frequently accompanied by psychiatric comorbidities, particularly anxiety disorders and alcohol use disorders [4].

Although antidepressants (ADs), psychotherapy, and their combination are all effective treatments for MDD in the short- and long-term [5], patients often prefer psychotherapy over ADs, partly because of concerns about adverse effects and a greater sense of autonomy and self-management [6].

Cognitive behavioral therapy (CBT) is one of the most extensively studied and widely implemented evidence-based psychotherapies for MDD in primary and specialized mental health care [5]. Compared with treatment as usual (TAU) or waiting-list control conditions, CBT demonstrates moderate to large treatment effects, whereas comparisons with other evidence-based psychotherapies generally show only small or nonsignificant differences [5]. Despite this strong evidence base, scaling up face-to-face CBT (ftfCBT) in routine care remains challenging. High treatment costs, long waiting lists, shortages of trained therapists, and limited reimbursement for CBT services—even in high-income countries—continue to restrict access to care [7]. As a result, a substantial global treatment gap persists for people with MDD [8].

Over the past 2 decades, internet-based cognitive behavioral therapy (iCBT) has been developed to address some of these challenges by offering an accessible, cost-effective, and scalable treatment format [9,10]. Numerous meta-analyses have demonstrated that self-guided and therapist-guided iCBT are effective in treating depression. Compared with nonactive controls and participants recruited from the general population, therapist-guided iCBT generally yields moderate to large effect sizes, whereas self-guided iCBT produces smaller, but still significant, effects [11]. Furthermore, a meta-analysis found no significant differences in depression outcomes between iCBT and ftfCBT among patients receiving routine mental health care. However, this meta-analysis by Hedman-Lagerlöf and colleagues [12] included only 5, mostly small-scale, randomized controlled trials (RCTs), highlighting the limited evidence available from routine care settings. Although these findings support the efficacy of iCBT, evidence from routine clinical practice remains limited. Together with the scarcity of implementation studies conducted under real-world conditions, this may partly explain the relatively slow uptake of iCBT for MDD in primary and specialized mental health care (with the exception of dedicated online treatment services).

In addition to the limited routine care evidence base, several barriers have been proposed to explain the slow implementation of iCBT. These include organizational factors (eg, limited managerial support), therapist-related concerns, patient-related barriers, and external factors such as data privacy regulations [13]. For example, therapists question whether findings from existing iCBT studies are generalizable to patients treated in routine clinical practice, who are often perceived to be more clinically complex and socially vulnerable than participants recruited from the general population because of greater symptom severity, higher rates of comorbidity, and greater socioeconomic disadvantage [14]. Therapists with little or no experience using iCBT often worry as well that delivering treatment remotely will hinder the development of a strong therapeutic alliance, despite evidence indicating that therapeutic alliance can be maintained [15,16]. Patients have identified barriers, also including the high level of motivation required to work independently and the reduced amount of face-to-face contact.

These implementation barriers have stimulated interest in blended treatment formats for MDD in routine care. Reviews have shown that blended CBT (bCBT) protocols vary with respect to their treatment composition (integrated or sequential), the role of the digital component (core or supplementary), and the technologies applied (internet, mobile apps, or both) [17,18]. By combining the strengths of face-to-face and digital treatment, bCBT may overcome several perceived limitations of stand-alone iCBT while retaining its advantages in terms of accessibility and efficiency. Recent studies have explored the acceptability and preferences of therapists and patients for bCBT in routine care. These studies indicate that many therapists and patients prefer bCBT over stand-alone iCBT if bCBT maintains the advantage of the clinical flexibility of face-to-face therapy and digitally supports patients’ self-management skills [19,20]. Few studies have evaluated the effectiveness of bCBT yet.

Nakao et al (2018) evaluated the effectiveness of bCBT in a small RCT (n=40), comparing bCBT plus standard psychiatric care with standard psychiatric care alone (waitlist) in patients with MDD who had not responded to AD medication. The study reported a large between-group effect in favor of bCBT (*d*=1.1) [21]. A smaller number of studies have yet clinically evaluated bCBT directly compared with ftfCBT for MDD. Kalde et al [22] (n=82) and Kooistra et al [23] (n=102), for example, reported no significant differences in depressive symptoms between treatment conditions at posttreatment. Although these findings are encouraging, neither study was powered to establish noninferiority. In addition, several bCBT trials have been described in published study protocols, with results awaited [24-26]. Consequently, robust evidence from large, pragmatic, multinational trials evaluating the clinical effectiveness and cost-effectiveness of bCBT in routine clinical practice remains limited.

To address this evidence gap, we conducted the European Comparative Effectiveness Research on Internet-based Depression Treatment (E-COMPARED) study across 9 European countries, evaluating an integrated form of bCBT in which face-to-face sessions were combined with structured internet- and smartphone-based modules within a single treatment protocol [27]. Within this protocol, face-to-face sessions were used for more in-depth therapeutic interactions, assessment of clinical needs, and shared decision-making, whereas the digital modules supported psychoeducation, homework assignments, symptom monitoring, and self-management.

We hypothesized that bCBT would be clinically noninferior to TAU for patients with MDD. In addition, we hypothesized that bCBT would reduce health care costs by requiring less therapist time while maintaining comparable clinical outcomes, thereby improving cost-effectiveness from a health care perspective. Demonstrating that bCBT is clinically noninferior and cost-effective would support its wider implementation, thereby improving access to evidence-based CBT for patients with MDD.

## Methods

### Study Design and Participants

We conducted a pragmatic 2-arm, open-label, multisite, randomized controlled clinical trial. We applied noninferiority design criteria, including an active comparator (TAU) and a preset noninferiority margin [28]. The E-COMPARED study was conducted across 8 European countries: France, Germany, the Netherlands, Poland, Spain, Sweden, Switzerland, and the United Kingdom, ensuring geographical coverage across Europe. Denmark was subsequently added due to the country’s emergent interest in bCBT. We conducted the RCTs in settings where both bCBT and TAU for MDD treatment could be provided. In Denmark, France, the Netherlands, and Switzerland, the study was conducted within specialized mental health services, while in Germany, Poland, Spain, Sweden, and the United Kingdom, it was conducted within primary care. The results were reported in accordance with the CONSORT (Consolidated Standards of Reporting Trials) guidelines for noninferiority trials [29]. We refer to the protocol for a detailed description of the study [27].

### Inclusion and Exclusion Criteria

The inclusion criteria were age ≥18 years, meeting *DSM-IV* (*Diagnostic and Statistical Manual of Mental Disorders* [Fourth Edition]) diagnostic criteria for MDD via the Mini International Neuropsychiatric Interview (MINI; excluding sections M [anorexia nervosa], N [bulimia nervosa], and P [antisocial personality disorder]) [30], and depressive symptoms (Patient Health Questionnaire-9 [PHQ-9] score ≥5) [31]. Patients were permitted to receive pharmacotherapy in addition to bCBT at baseline or during treatment, if deemed necessary by a health care professional, in line with routine clinical practice. At the 12-month follow-up, phone-based MINI interviews were conducted by independent, trained interviewers who were masked to the assigned group. The exclusion criteria included currently being at high risk of suicide (MINI section C), psychiatric comorbidity (substance dependence, bipolar disorder, psychosis, and obsessive-compulsive disorder), currently undergoing psychological treatment for depression, language comprehension barriers, lack of a stable internet connection or access to a computer, and either the absence of a compatible smartphone or an unwillingness to loan one for bCBT.

### Procedures

Recruitment took place between February 2015 and December 2016; data collection closed on December 31, 2017. Patients were approached by mental health professionals in health care settings, who then referred them to the research team for eligibility assessment. In Germany and Sweden, recruitment leaflets were placed in general practitioner waiting rooms. Sweden also used social media platforms to recruit patients who had contacted primary care services about depressive complaints. Multimedia Appendix 1 provides an overview of the recruitment procedures for each country, along with any additional procedures carried out.

### Ethical Considerations

Ethical approval and trial registrations were obtained at the national level. Participants provided written informed consent, authorizing the E-COMPARED partners to share and analyze the anonymized dataset and to report the results in publications. Participants did not receive compensation for study participation. Ethical approval for the trials has been obtained locally in each country: Ethics Committee of the Region of Southern Denmark (S-20150150); France: Comité de protection des personnes, Île de France V (15033-n 2015-A00565-44); Germany: Ethik Kommission DGPsychologie, Universität Trier (MB 102014); The Netherlands: METC VUMC (2015.078); Poland: Komisja ds. Etyki Badań Naukowych (10/2014); Spain: Comisión Deontológica/Comité Ético de Investigación en Humanos (H1414775276823); Sweden: Regionala etikprövningsnämnden (2014/428-31); Switzerland: Kantonale Ethikkommission Bern (001/2015); and United Kingdom: NRES Committee London-Camden and King’s Cross (15/LO/0511).

### Randomization and Masking

After the baseline assessment, randomization of patients was conducted centrally at VU Amsterdam (Netherlands) by an independent researcher (EK) using automated Random Allocation Software (Department of Anaesthesia, Isfahan University of Medical Sciences). The allocation, stratified by country, was 1:1. Block randomization was used with variable block sizes, ranging from 2 to 14 allocations per block. In the United Kingdom, the process was outsourced to a local independent researcher for logistical reasons, which required the randomization outcome to be available immediately. Furthermore, stratification by recruitment location was applied at the national level for France (11 centers), Switzerland (3 centers), the Netherlands (3 centers), and Sweden (2 cities). Researchers and clinicians were blinded to the randomization scheme, but blinding of treatment allocation was impossible because both groups were evident to the therapists and patients.

### Sample Size Calculation

RCTs can determine whether a treatment effect is statistically significant and quantify its magnitude, but they do not necessarily capture the clinical relevance of the findings. Several approaches exist to assess clinical importance. In this study, we adopt the minimal clinically important difference from the patient perspective, as proposed by Cuijpers and colleagues [32], who identified a threshold of Cohen *d*=0.24. Given our noninferiority design, we applied a more conservative margin of *d*=0.20 for the primary outcome (PHQ-9). Under this criterion, bCBT is considered noninferior to TAU in clinical terms if the upper bound of the confidence interval does not exceed this predefined margin (1-sided tested). The required sample size was 1052 patients; to allow for dropouts, we aimed to enroll 1200 patients. This calculation was based on 90% power (type II error ≤0.10) and the requirement that the upper bound of the 2-sided 95% CI would not exceed the prespecified noninferiority margin.

### bCBT for MDDs

The bCBT protocols used in this study were based on ftfCBT protocols for MDD. The bCBT protocols integrated face-to-face sessions with structured internet-based treatment modules and ecological momentary assessments (EMAs) via smartphone to monitor daily mood, cognition, activity, social interaction, and sleep. During these face-to-face sessions, the core principles of CBT were applied, alongside mandatory internet modules at all participating sites (Table 1). These mandatory modules focused on psychoeducation, cognitive restructuring, behavioral activation, and relapse prevention, as well as homework assignments. These internet-based modules were discussed in subsequent face-to-face sessions. The mandatory internet modules comprised 75% of the total number of modules. Sites also had the option to add extra modules (25%), such as emotional regulation or coping skills training, to facilitate adaptation to setting-specific practices, while maintaining protocol standardization across sites. At least one-third of the total number of sessions were to be held face-to-face, while another one-third were to be held online to ensure sufficient face-to-face and online modules.

**Table 1.** Overview of blended cognitive behavioral therapy applied within each country by platform, setting, duration, number of face-to-face and internet sessions, sequencing and mandatory internet modules (X), and additional modules.

Country	Platform	Setting	Duration	Number of face-to-face and internet modules	Sequencing	Mandatory internet modules (X)^a^ and additional modules
Germany	Moodbuster	PC^b^	12 weeks	6/10	Alternate	X PS^c^/PEX^d^
Poland	Moodbuster	PC	8 weeks	7/6	Alternate	X PS/PEX
Sweden	Iterapi	PC	10 weeks	4/6	Alternate	X Other
United Kingdom	Moodbuster	PC	11 weeks	6/5	Alternate	X PS/PEX
Spain	Smiling is Fun	PC/SMHC^e^	10 weeks	3/8	1-4-1-4-1	X PS/PE^f^/CS^g^
Denmark	NoDep	SMHC	12 weeks	12/7	Alternate	X other
France	Moodbuster	SMHC	16 weeks	8/8	Alternate	X PS/PEX
Netherlands	Moodbuster	SMHC	20 weeks	10/10	Alternate	X PS/PEX
Switzerland	Deprexis	SMHC	18 weeks	9/9	Alternate	X Other

a X: mandatory modules.

b PC: primary care.

c PS: problem-solving.

d PEX: physical exercise.

e SMHC: specialized mental health care.

f PE: psychoeducation.

g CS: coping skills.

Table 1 summarizes, by country, the setting, duration, intensity, the alternation between face-to-face sessions and online modules, and additional modules for the bCBT group. As we followed routine care procedures, 5 countries used their own bCBT platforms: Denmark, the Netherlands, Spain, Sweden, and Switzerland. Four countries—France, Germany, Poland, and the United Kingdom—implemented a translated version of the Dutch Moodbuster platform for their patients because no bCBT platforms were available in these countries. All these platforms complied with General Data Protection Regulation requirements [33]. bCBT was delivered within primary care in 4 countries, while in Spain, it was provided both within primary care and outpatient specialized mental health care due to constraints within the original specialized mental health care setting. In the other 3 countries, it was delivered within specialized outpatient mental health care settings. bCBT began with a face-to-face session in all the countries. Within primary care, the treatment duration of bCBT ranged from 6 to 13 weeks, comprising 3-7 face-to-face sessions and 5-10 internet modules. Within specialized mental health care services, the treatment duration was longer, typically from 12 to 20 weeks, with 8-12 face-to-face sessions and 7-10 internet modules. All countries alternated face-to-face sessions with online modules, except for Spain (which used 2 face-to-face sessions, 4 internet modules, and a final face-to-face session). The total number of ftfCBT sessions ranged from 3 to 6 in primary care settings and from 8 to 12 in specialized mental health care settings, and sessions were conducted in accordance with guidelines for treating moderate to severe MDD [34]. These face-to-face sessions lasted between 45 and 60 minutes each. The internet modules required 30-45 minutes of patients’ time and needed 3-5 minutes daily to answer the EMA questions. The therapists used the platforms to monitor their patients’ progress and to communicate securely with them. They were required to spend approximately 15 minutes each week per patient to review their online homework assignments. CBT therapists comprised licensed professionals and supervised psychology trainees; supervision by a licensed psychologist specializing in CBT applied only in primary care settings.

### Treatment as Usual

All participants in the control group received active treatment. TAU comprised routine care for patients with MDD in the treatment settings from which they were recruited (primary care or specialized mental health care). Six countries delivered ftfCBT within routine care (Denmark, France, the Netherlands, Poland, Switzerland, and the United Kingdom). In Sweden, Germany, and partly in Spain, TAU consisted of generic general practitioner care, which might have included watchful waiting, referral to a mental health specialist, psychotherapy (in Sweden and Germany), pharmacological treatment, or a combination of these. In the ftfCBT settings, therapists’ professional qualifications were similar to those in the bCBT groups.

### Outcomes, Measures, and Time Points

All measures were self-reported and completed online (except for the MINI, which was assessed by independent interviewers blinded to the groups). Primary and secondary outcomes (including costs) were assessed at baseline and at the 3, 6, and 12-month follow-up periods. The MINI was assessed only at baseline and at the 12-month follow-up. We followed routine care RCT protocols in the participating countries, which resulted in primary care studies being shorter in duration and lower in intensity than those conducted in specialized mental health care settings. Posttreatment assessments meant that all participants could have completed their treatment 6 months after baseline. To enable an overall comparison, we therefore defined the 3-month assessment as a during-treatment measurement, the 6-month assessment as a posttreatment measurement, and the 12-month assessment as a follow-up measurement. The outcomes and time points are presented in Table 2. The reliability of the questionnaires is presented in Multimedia Appendix 2. Cronbach α ranged from 0.70 to 0.98, indicating good overall reliability.

**Table 2:** Overview of measures and time points.

Variable	Measures	Baseline	3 months	6 months	12 months
Questions asked to patients					
Demographics	N/A^a^	✓	N/A	N/A	N/A
Diagnostic interview	MINI^b^	✓	N/A	N/A	✓
Depressive symptoms	PHQ-9^c^ and QIDS-SR16^d^	✓	✓	✓	✓
Quality of life	EQ-5D-5L	✓	✓	✓	✓
Societal and health care costs	TiC-P^e^	✓	✓	✓	✓
Treatment preference	N/A	✓	N/A	N/A	N/A
Patient expectancy and credibility	CEQ^f^	✓	N/A	N/A	N/A
Working alliance	WAI-SF-C^g^	N/A	✓	N/A	N/A
Technology alliance	WAI-TECH-SF^h^	N/A	✓	N/A	N/A
Patient satisfaction	CSQ^i^	N/A	✓	N/A	N/A
Satisfaction with the online program	SUS^j^	N/A	✓	N/A	N/A
Questions asked to therapists					
Working alliance	WAI-SF-Therapists	N/A	✓	N/A	N/A

a N/A: not applicable.

b MINI: Mini International Neuropsychiatric Interview.

c PHQ-9: Patient Health Questionnaire-9.

d QIDS-SR 16: Quick Inventory of Depressive Symptomatology Self-Report-16.

e TiC-P: Trimbos/iMTA questionnaire for Costs associated with Psychiatric illness.

f CEQ: Credibility and Expectancy Questionnaire.

g WAI-SF-C: Working Alliance Inventory-Short Form–Client.

h WAI-TECH-SF: Working Alliance Inventory for Online Interventions-Short Form.

i CSQ: Client Satisfaction Questionnaire.

j SUS: System Usability Scale.

### Primary Outcome

The PHQ-9 was the primary outcome at posttreatment. It has demonstrated good psychometric properties in both primary and specialized care populations [30,35,36]. The total score ranges from 0 to 27, with 0‐4 indicating no to minimal depression, 5‐9 indicating mild depression, 10‐14 indicating moderate depression, 15‐19 indicating moderately severe depression, and 20‐27 indicating severe depression. Paper-based and online versions of the PHQ-9 yield comparable psychometric properties [37]. The PHQ-9 was also used to assess reliable deterioration. Participants were classified as having reliably deteriorated when the increase in PHQ-9 score exceeded the Reliable Change Index (RCI) threshold (|RCI|>1.96) [38]. See Multimedia Appendix 3 for a detailed description of the deterioration calculation.

### Secondary Outcomes

The Quick Inventory of Depressive Symptomatology Self-Report-16 (QIDS-SR 16) was used as a second measure to assess the level of depressive complaints [39]. The MINI [31] was used to evaluate MDD and comorbid anxiety disorders at baseline and at the 12-month follow-up to assess depression-free episodes (remission).

### Additional Outcomes

#### Overview

Patient treatment expectations were measured with the Credibility and Expectancy Questionnaire (maximum score of 27 [40]) at baseline only. The remaining 4 outcomes were assessed during treatment, 3 months after the baseline assessment. Treatment satisfaction was assessed using the Client Satisfaction Questionnaire-8 (CSQ-8; range 8-32 [41]), and satisfaction with the online platforms was evaluated using the System Usability Scale (SUS) [42]. The therapeutic alliance between patients and therapists was measured using the short version of the Working Alliance Inventory, which comprises the Bond, Task, and Goal subscales, and a composite score (Working Alliance Inventory-Short Form [WAI-SF]) for patients [43] and therapists [44]. For descriptive purposes, patients’ and therapists’ ratings were transformed into a 0-5 range for each component separately (as well as for the composite score). Patients’ alliance with online platforms (experimental group only) was evaluated using the Working Alliance Inventory–Technology (Working Alliance Inventory for Online Interventions-Short Form [WAI-TECH-SF] [45], derived from the WAI-SF). Demographic variables such as age, sex, education, marital status, income, and employment, along with mental health treatment history, were assessed only at baseline. Patients’ preference for bCBT or TAU was determined using a simple yes/no/no-preference question.

#### Cost Measures

Costs were evaluated from the health care perspective, based on health care and patient costs, using the TiC-P (Trimbos/iMTA questionnaire for Costs associated with Psychiatric illness) questionnaire at baseline and at 3, 6, and 12 months [46]. To avoid cost discrepancies due to varying prices across countries, resource usage was assessed using standard Dutch costs [47]. For reference, the average 2017 exchange rate was €1=US $1.1297 (European Central Bank).

### Data Collection and Analyses

Each participating country collected data in accordance with a predefined data codebook, which was then uploaded to a centralized database (Research Department of GGZ inGeest Mental Health Care). The encrypted data, free of identifiable patient and therapist information, complied with General Data Protection Regulation regulations [33].

### Statistical Analyses

Analyses were based on the intention-to-treat (ITT) principle. We initially considered a per-protocol analysis, as ITT approaches in noninferiority trials may dilute between-group differences by including nonadherent participants, potentially biasing results toward noninferiority [48]. However, we ultimately adopted an ITT analysis because per-protocol can compromise the benefits of randomization and introduce selection bias. In addition, ITT better reflects routine clinical practice, where nonadherence is common, thereby enhancing generalizability to routine care settings. It also captures real-world effectiveness, aligns with health economic evaluation objectives, and accounts for variability in adherence and outcomes.

Descriptive statistics were used to assess baseline characteristics of the participants (Table 3). Multiple imputation was conducted using chained equations with predictive mean matching until the loss of efficiency was less than 5%, resulting in 20 complete datasets [49]. Linear mixed modeling [50] estimated the mean levels of the outcome variable for the TAU group and the intervention effects at 3 time points, using 3 time dummies (3-, 6-, and 12-month measurements) and 3 time dummy-by-group interaction terms; hypothesis tests for these interaction effects were conducted 1-sided, in line with the noninferiority framework. Alongside these fixed effects (for the main estimates), the model included random effects (random intercepts and time slopes for countries) and allowed for correlated residuals (for measurements within individuals). Missing observations for the CSQ and SUS were not imputed, as the answers to these questions were highly subjective. This reduces the risk of bias due to incorrect imputation [51]; AD use at the 3-, 6-, and 12-month assessments was also not imputed. Estimated intervention effects (EE) were presented on the corresponding scales for the 4 outcomes. Cohen *d* and 95% CIs [52] were used to quantify between-group treatment effect sizes for both the PHQ-9 and the QIDS. Effect sizes were classified as very small (*d*<0.2), small (0.2-0.5), medium (0.5-0.8) or large (>0.8). MINI-D (Depression) and MINI-A (Anxiety) were presented as log-odds ratios using the Hasselblad and Hedges logit method [53]. A “leave-one-out” sensitivity analysis assessed the robustness of the overall effect by systematically excluding one country at a time and recalculating the effect size for the primary outcome [54]. To assess the impact of baseline AD use on treatment outcomes during treatment and follow-ups, we extended the linear mixed modeling by adding a third factor (AD use: yes/no). This allowed estimation of marginal mean PHQ-9 scores at different time points for the bCBT and TAU groups within strata of AD use, as well as effect sizes. We calculated a reliable deterioration in the PHQ-9 [38] during treatment (3 months), posttreatment (6 months), and at the 12-month follow-up point. The nonimputed dataset assessed the number of patients who deteriorated, while the log ratios and corresponding effect sizes were estimated using generalized linear (logistic) mixed models. Stata (version 14.2; StataCorp LLC) was used to conduct the analyses.

**Table 3.** Baseline sociodemographic patient characteristics.

Characteristics	bCBT^a^	TAU^b^	Total
Patients, n	423	412	835
Age (years), mean (SD)	39.52 (13.27)	38.84 (13.09)	39.17 (13.18)
Sex (female), n %	282 (66.67)	281 (68.20)	563 (67.42)
Marital status, n (%)			
Single	142 (33.57)	132 (32.04)	274 (32.81)
Divorced	55 (13.00)	39 (9.47)	94 (11.26)
Widowed	2 (0.47)	4 (0.97)	6 (0.72)
Living together	87 (20.57)	103 (25.00)	190 (22.75)
Married	137 (32.39)	134 (32.52)	271 (32.46)
Education, n (%)^c^			
Low	54 (12.77)	60 (14.56)	114 (13.65)
Middle	156 (36.88)	145 (35.19)	301 (36.05)
High	213 (50.35)	207 (50.24)	420 (50.30)
Employed, n (%)	N=418	N=409	N=827
Yes	248 (59.33)	263 (64.30)	511 (61.79)
No	170 (40.67)	146 (35.70)	316 (38.21)
MINI^d^, n (%)			
Major Depressive Disorder	423 (100)	412 (100)	835 (100)
Anxiety Disorder	221 (52.25)	199 (48.30)	420 (50.30)
PHQ-9^e^, mean (SD)	15.36 (4.82)	15.21 (4.53)	15.28 (4.67)
QIDS^f^, mean (SD)	14.60 (4.40)	14.65 (4.23)	14.63 (4.31)
Antidepressant use, n/N (%)	168/417 (40.29)	184/406 (45.32)	352/823 (42.77)
Treatment preference, n (%)			
bCBT	241 (56.97)	230 (55.83)	471 (56.41)
TAU	73 (17.26)	64 (15.53)	137 (16.41)
No preference	109 (25.77)	118 (28.64)	227 (27.18)
CEQ_cred, mean (SD)^g^	19.45 (4.73)	16.97 (6.13)^h^	18.32 (5.55)
CEQ_exp, mean (SD)^g^	17.57 (4.96)	15.45 (6.09)^h^	16.60 (5.61)
UTILNL^i^, mean (SD)	0.58 (0.23)	0.57 (0.24)	0.57 (0.23)
Health care costs (€, TiC-P^j^, 4 weeks), mean (SE)^j^	1172 (185)	1338 (183)	1255 (12.74)

a bCBT: blended cognitive behavioral therapy.

b TAU: treatment as usual.

c Data collected were regarding what would be considered low, middle, and high levels of education in each setting.

d MINI: Major Depressive Disorder, Anxiety Disorder.

e PHQ-9: Patient Health Questionnaire-9.

f QIDS: Quick Inventory of Depressive Symptomatology Self-Report.

g CEQ cred and CEO exp: Credibility and Expectancy Questionnaire [40].

h *P* <.001.

i UTILNL: scores, Netherlands [55].

j TiC-P: Trimbos/iMTA questionnaire for Costs associated with Psychiatric illness [46].

### Cost-Effectiveness Analyses

Cost-effectiveness analyses were conducted from the health care perspective, as this perspective best aligned with our hypothesis. This perspective is also recommended by the National Institute for Health and Care Excellence in the United Kingdom [56]. Health-related quality of life for the cost-effectiveness analyses was measured using the EQ-5D-5L [57]. EQ-5D-5L health states were converted into utility scores using the Dutch tariff [58], and quality-adjusted life years (QALYs) were calculated by multiplying utilities by patient time spent in each health state, with linear interpolation applied between transitions.

Within the multiple imputation procedure, predictive mean matching was used to account for the skewed distribution of cost data. Mixed-effects regression models with repeated measurements clustered within participants were used to estimate differences in overall PHQ-9 scores and the number of MDD cases prevented over the 12-month follow-up period. Differences in QALYs and costs were analyzed using linear regression models.

Incremental cost-effectiveness ratios (ICERs) were calculated by dividing the difference in costs between the 2 conditions by the corresponding difference in effects. Statistical uncertainty surrounding the ICERs was assessed using bias-corrected and accelerated bootstrapping with 5000 replications and visualized in cost-effectiveness planes. Cost-effectiveness acceptability curves were constructed to estimate the probability that bCBT was cost-effective compared with TAU across a range of willingness-to-pay (WTP) thresholds. The WTP threshold represents the maximum amount society is willing to pay for 1 additional unit of health gain. No established WTP threshold exists for improvements in PHQ-9 scores. For QALYs, however, a maximum WTP threshold of €50,000 per QALY gained was applied, in accordance with the Dutch Health Care Institute’s recommendation for interventions targeting conditions with an estimated disease burden of 0.48. Finally, a sensitivity analysis was conducted in which differences in costs and effects were adjusted for potential confounders, including baseline health care costs, age, sex, and treatment setting (primary vs secondary care) [55,56]. Analyses were conducted in Stata (version 14.2), and reporting adhered to the CHEERS (Consolidated Health Economic Evaluation Reporting Standards) [59].

## Results

### Overview

**Figure 1.**
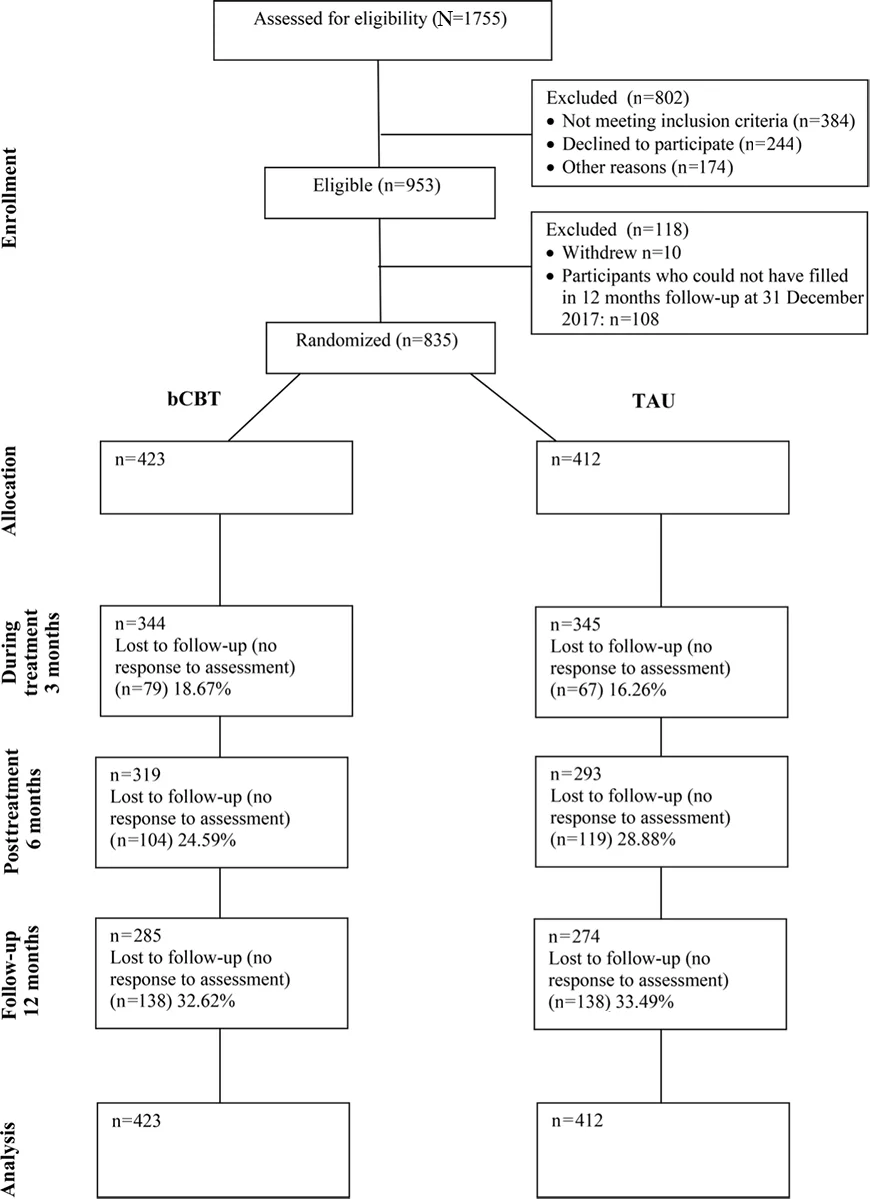
Flow diagram of participants through the trial based on intention-to-treat (CONSORT [Consolidated Standards of Reporting Trials]). bCBT: blended cognitive behavioral therapy; TAU: treatment as usual.

Figure 1 illustrates the study’s participant flow. Of 1755 participants assessed for eligibility, 953 started treatments, of whom 118 were excluded from our analysis. Ten participants withdrew before randomization, and for 108, the 12-month assessment would be beyond December 31, 2017 (the funder-determined time frame). Thus, the ITT sample included 835 participants, with 423 in bCBT and 412 in TAU. The total study dropout rates for the sample were 17.5% at 3 months after baseline assessment, 26.7% after 6 months, and 33% after 12 months.

Table 3 summarizes the participants’ baseline sociodemographic, clinical, and cost profiles. Multimedia Appendix 4 presents participant counts and baseline characteristics for each country. The mean age of the total group was 39.17 (SD 13.18) years, with 67% female and half of the sample having a high level of education. The employment rate was 61.80%. The mean PHQ-9 score for the total group was 15.28 (SD 4.7), indicating moderately severe symptoms. Over half (420/835, 50.30%) of the participants had a comorbid anxiety disorder. At baseline, on average, 42.77% (352/823) of the participants used ADs, with no significant differences between the 2 groups. This percentage remained stable over time, averaging 42.65% (351/823) at 3 months, 38.40% (316/823) at 6 months, and 40.70% (335/823) at 12 months, with nonsignificant differences between the groups. At prerandomization, 56.41% (471/835) of the participants preferred bCBT, 16.41% (137/835) preferred TAU, and the remainder reported no preference. The bCBT group scored significantly higher on the credibility and expectancy scales for treatment than the TAU group, indicating moderate to high levels on both measures. Mean health care costs per patient 4 weeks prebaseline were €1172 (SE €185) for the bCBT group and €1338 (SE €183) for the TAU group.

### Primary Outcomes

**Figure 2.**
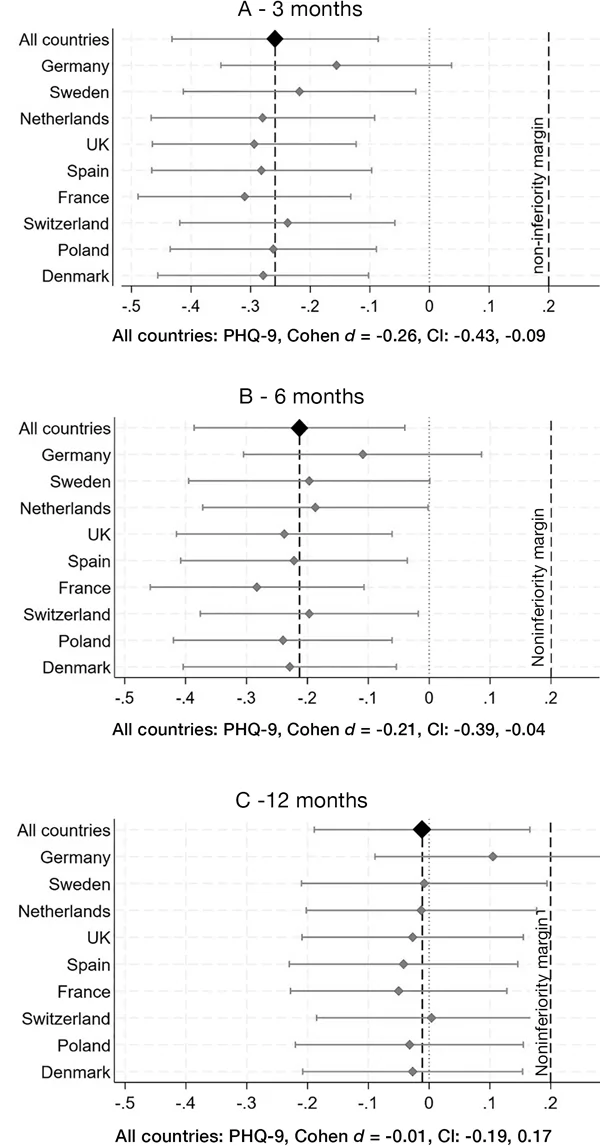
Leave-one-out meta-analysis (sensitivity analysis). Primary outcome PHQ-9: effect sizes and confidence intervals at (A) 3 months (during treatment), (B) 6 months, and (C) 12 months (N=835). PHQ-9: Patient Health Questionnaire-9.

Table 4 and Figure 2 show that, for the primary outcome (PHQ-9), bCBT was noninferior to TAU at 3 (Figure 2A), 6 (Figure 2B), and 12 (Figure 2C) months, as all upper bounds of the confidence intervals lay within the noninferiority margin of ≥0.20. A “leave-one-out” sensitivity analysis also showed noninferiority of the primary outcome at the 3- and 6-month assessments. However, at 12 months, the “leave-one-out” analysis revealed that excluding Germany yields inferior results. This indicates that the overall effect estimate is sensitive to this omission and suggests potential heterogeneity across settings. Together, these findings highlight uncertainty in the 12-month results and suggest that the observed effects may be context-dependent.

**Table 4:** Estimated intervention effects and effect sizes on both the primary outcome (PHQ-9)^a^ and secondary outcomes (QIDS^b^, MINI^c^ Depression, and MINI Anxiety), based upon both 1-stage models with random time slopes based upon multiple imputed data (N=835).

Outcomes	3 months	6 months	12 months
Primary outcome: PHQ-9			
EE^d^ (SE)	−1.21 (0.41); *z*=−2.939; *P*=.003	−1.00 (0.41); *z*=−2.411; *P*=.02	−0.05 (0.42); *z*=−0.124; *P*=.90
ES^e^ (CI)	*d*=−0.26 (−0.43 to −0.09)	*d*=−0.21 (−0.39 to −0.04)	*d*=−0.01 (−0.19 to 0.17)
Secondary outcomes			
QIDS-16			
EE (SE)	−1.15 (0.46); *z*=−2.489; *P*=.01	−0.33 (0.39); *z*=−0.831; *P*=.41	0.14 (0.42); *z*=0.340; *P*=.73
ES (CI)	*d*=−0.27 (−0.48 to −0.06)	*d*=−0.08 (−0.25 to 0.10)	*d*=0.03 (−0.16 to 0.22)
MINI-Depression			
OR^f^ (CI)	N/A^g^	N/A	0.67 (0.45 to 0.99)
MINI-Anxiety			
OR (CI)	N/A	N/A	0.78 (0.46 to 1.32)

a PHQ-9: Patient Health Questionnaire-9.

b QIDS: Quick Inventory of Depressive Symptomatology.

c MINI: Mini International Neuropsychiatric Interview.

d EE: estimated intervention effects.

e ES: effect sizes.

f OR: odds ratio.

g N/A: not applicable.

### Secondary Outcomes

#### Overview

Noninferiority of bCBT is also shown in Table 4 for the secondary QIDS outcome at 3 months during treatment (*d*=−0.27, 95% CI −0.48 to −0.06) and at posttreatment (6 months) (*d*=−0.08, 95% CI −0.25 to 0.10) but not at 12 months’ follow-up (95% CI −0.16 to 0.22). The bCBT group also had a lower risk of MDD than the TAU group at the 12-month follow-up (odds ratio [OR] 0.67, 95% CI 0.45-0.99) for anxiety disorders; this difference was not significant (OR 0.78, 95% CI 0.46-1.32).

**Figure 3.**
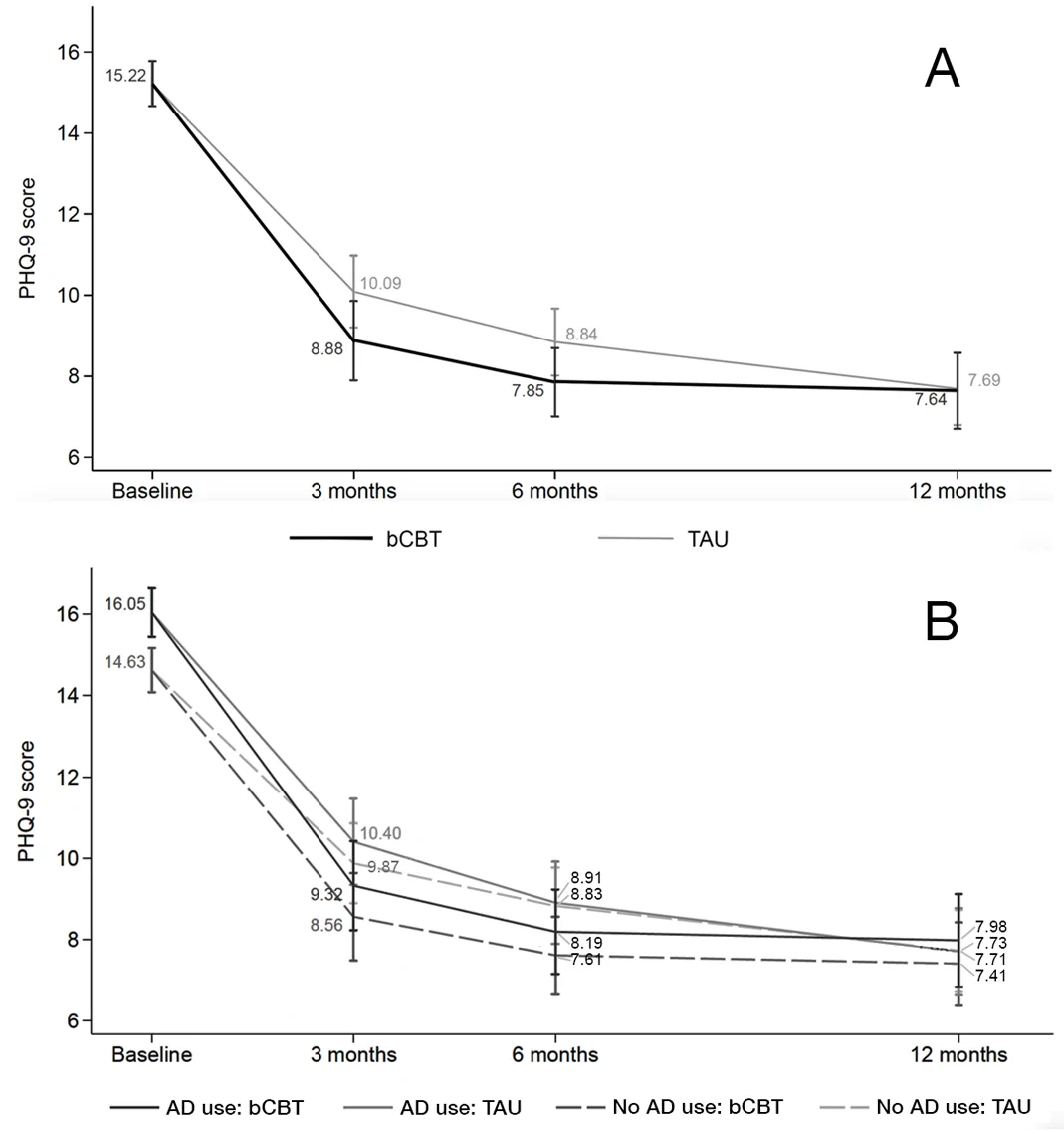
Estimated marginal means PHQ-9 scores at various time points for the bCBT and TAU group (A) and for strata of AD use (B). AD: antidepressant; bCBT: blended cognitive behavioral therapy; CBT: cognitive behavioral therapy; PHQ-9: Patient Health Questionnaire-9; TAU: treatment as usual.

As shown in Table 4 and Figure 3A, both groups exhibited significant within-group changes in the primary outcome, with a rapid decrease leading up to posttreatment at 6 months. Differences were small but significant for bCBT over TAU during treatment (3 months, *d*=−0.26, 95% CI −0.43 to −0.09; *P*=.003) and at 6 months posttreatment (*d*=−0.21, 95% CI −0.39 to −0.04; *P*=.016), with a nonsignificant difference between the groups at the 12-month follow-up.

A subgroup analysis (Table 5 and Figure 3B) was conducted to estimate the impact of baseline AD versus non-AD use on the primary outcome (PHQ-9) in bCBT versus TAU. At baseline, the AD groups had a 1.42-point higher PHQ-9 score than the non-AD user groups, indicating that this group experienced more severe depressive symptoms. AD patients exhibited a greater reduction in depressive symptoms than non-AD users, most likely because their higher baseline scores left more room for improvement (Figure 3). When comparing the impact of AD use and non-AD use on the primary outcome across the 2 groups, no significant differences were found at any of the 3 time points (Table 5, AD vs no AD at baseline, bCBT compared with TAU).

**Table 5.** Estimated marginal mean PHQ-9^a^ scores at various time points for the bCBT^b^ and TAU^c^ groups and by strata of AD^d^ use.

	Difference in EMM^e^ (95% CI)	Difference in Cohen *d* (95% CI)	*P* value
No AD at baseline bCBT-TAU			
3 months	−1.31 (−2.35 to −0.28)	−0.28 (−0.50 to −0.06)	.01
6 months	−1.21 (−2.28 to −0.15)	−0.26 (−0.49 to −0.03)	.02
12 months	−0.32 (−1.41 to 0.77)	−0.07 (−0.30 to 0.16)	.56
AD at baseline bCBT-TAU			
3 months	−1.08 (−2.26 to 0.10)	−0.23 (−0.48 to 0.02)	.07
6 months	−0.71 (−1.95 to 0.52)	−0.15 (−0.42 to 0.11)	.26
12 months	0.27 (−0.97 to 1.52)	0.06 (−0.21 to 0.32)	.66
AD versus no AD at baseline bCBT-TAU			
3 months	0.23 (−1.26 to 1.73)	0.05 (−0.27 to 0.37)	.76
6 months	0.50 (−1.12 to 2.12)	0.11 (−0.24 to 0.45)	.54
12 months	0.59 (−1.03 to 2.22)	0.13 (−0.22 to 0.47)	.47

a PHQ-9: Patient Health Questionnaire-9.

b bCBT: blended cognitive behavioral therapy.

c TAU: treatment as usual.

d AD: antidepressant.

e EMM: estimated marginal means.

Using the nonimputed dataset for the entire sample, the overall percentage of patients who deteriorated was low: 4.8% (33/688) at the 3-month assessment, 4.6% (28/609) at the 6-month assessment, and 4.1% (23/561) at the 12-month assessment. There were no significant differences in EE on reliable deterioration between the groups at all 3 time points (OR and Cohen *d*). However, the estimated multilevel logistic model for deterioration yielded unreliable estimates, most likely due to the small number of patients who deteriorated.

#### Acceptance and Working Alliance

Table 6 presents results for several acceptance and working alliance measures. Patients in the bCBT group scored significantly higher on treatment satisfaction (CSQ-8) than those in the TAU group, with a mean of 25.44 (SD 4.93) at the 3-month assessment. This score falls between “somewhat satisfied” (n=24) and “very satisfied” (n=32), whereas the TAU group scored below the 24 threshold (mean 21.34, SD 5.94). Only the bCBT group (patients) rated the technological platforms’ usability, with a mean SUS score of 40.16 (SD 7.35), below the average of 68. In contrast, the technical alliance (WAI-TECH-SF-C48), also rated only by the bCBT group (patients), was rated more positively, with a mean of 57.12 (SD 16.87) on a scale of 12‐84. Higher scores indicate a better quality of the technical alliance.

**Table 6:** Mean scores for user satisfaction and working and technical alliances by bCBT^a^ and TAU^b^.

	bCBT	TAU
CSQ^c^, N (mean, SD)	203 (25.51, 4.71)	194 (21.34, 5.94)^d^
SUS^e^, N (mean, SD)	324 (40.16, 7.35)	N/A^f^
WAI-SR-C^g^, composite, N (mean, SD)	265 (3.97, 0.77)	240 (3.41, 0.99)^d^
WAI-SR-C task, N (mean, SD)	265 (3.86, 0.84)	240 (3.24, 1.13)^d^
WAI-SR-C bond, N (mean, SD)	265 (4.19, 0.83)	240 (3.78, 1.03)^d^
WAI-SR-C goal, N (mean, SD)	265 (3.86, 0.84)	240 (3.21, 1.10)^d^
WAI-TECH-SF-C^h^, N (mean, SD)	297 (57.12, 16.87)	N/A
WAI-SR-Therap^i^ composite, N (mean, SD)	243 (3.84, 0.70)	216 (4.09, 0.64)^d^
WAI-SR-Therap task, N (mean, SD)	243 (3.54, 0.87)	216 (3.88, 0.79)^d^
WAI-SR-Therap bond, N (mean, SD)	243 (4.33, 0.63)	216 (4.41, 0.60)
WAI-SR-Therap goal, N (mean, SD)	243 (3.65, 0.90)	216 (3.99, 0.78)^d^

a bCBT: blended cognitive behavioral therapy.

b TAU: treatment as usual.

c CSQ: Client Satisfaction Questionnaire.

d *P*<.001.

e SUS: System Usability Scale.

f N/A: not applicable.

g WAI-SR-C: Working Alliance Inventory-Short Revised–Client.

h WAI-TECH-SF-C: Working Alliance Inventory for Online Interventions-Short Form–Client.

i WAI-SR-Therap: Working Alliance Inventory-Short Revised–Therapists.

Patients in bCBT and TAU rated the working alliance as good, with mean overall composite scores of 3.97 (SD 0.77) in the bCBT group and 3.41 (SD 0.99) in the TAU group, out of 5. However, patients in the bCBT group rated the therapeutic alliance (WAI-SR-C) significantly higher (*P*<.001) than those in the TAU group, both for the composite score and for the task, bond, and goal components. The therapists also rated the working alliance as good, but those in the TAU group rated it significantly higher (mean 4.09, SD 0.64; *P*<.001) than those in the bCBT group (mean 3.84, SD 0.70). They also scored significantly higher on the task (*P*<.001) and goal (*P*<.001) components of the WAI-SR-T in TAU compared with bCBT but not on the bond scale.

### Costs

Table 7 presents several disaggregated health care cost categories and total health care costs per patient for bCBT and TAU at the 12-month assessment. These costs did not differ significantly between the 2 groups, except for home care costs, which were significantly higher for TAU than for bCBT. However, the average total health care costs per patient were slightly higher for the bCBT group than for the TAU group (estimated mean difference per patient €315, 95% CI −582 to 1163). This difference was primarily driven by the estimated bCBT intervention costs.

**Table 7.** Estimated mean (SE) costs (euros) per patient for bCBT^a^ and TAU^b^, and corresponding differences (95% CI) at 12-month follow-up point (N=835).

Cost category	bCBT (€/SE)	TAU (€/SE)	Difference (95% CI)
Intervention costs	274 (6)	0 (0)	N/A^c^
Primary care costs	440 (31)	525 (49)	−85 (−213 to 7)
Mental health care costs	1517 (159)	1458 (142)	59 (−318 to 455)
Complementary care costs	74 (13)	55 (13)	18 (−16 to 48)
Secondary care costs	1131 (230)	745 (129)	386 (−25 to 900)
Home care costs	687 (96)	1029 (200)	−342 (−867 to −8)
Mental health medication costs	35 (3)	33 (3)	3 (−4 to 9)
Total health care costs	4159 (345)	3845 (331)	315 (−582 to 1163)

a bCBT: blended cognitive behavioral therapy.

b TAU: treatment as usual.

c Not applicable.

### Cost-Effectiveness Analyses

Table 8 presents the cost-effectiveness analysis results at the 12-month follow-up from a health care perspective. The mean overall change in PHQ-9 score over the 12-month period was small but significantly higher for bCBT than for TAU. Within the main analysis, the ICER for the PHQ-9 score was €643 per point of improvement, meaning that to gain a single point of improvement in the PHQ-9 score, an investment of €643 is required for bCBT compared with TAU.

**Table 8.** Multiple imputed, pooled cost-effectiveness outcomes for bCBT^a^ compared with TAU^b^ (euros) at the 12-month follow-up point^c^.

Outcome	ΔC^d^ (95% CI) (€)	ΔE^e^ (95% CI)	ICER^f^ (€)	CE^g^ plane			
				NE^h^	SE^i^	SW^j^	NW^k^
Main analysis							
PHQ-9^l^	315 (−533 to 1168)	0.49 (0.01 to 0.97)	643	73%	25%	0%	2%
MINI^m^	315 (−533 to 1168)	0.07 (0.01 to 0.14)	4381	74%	25%	0%	1%
QALY^n^	315 (−533 to 1168)	0.01 (−0.02 to 0.04)	36,729	51%	21%	4%	24%
Adjusted analysis							
PHQ-9	421 (−319 to 1161)	0.49 (0.02 to 0.97)	852	82%	16%	0%	2%
MINI	421 (−319 to 1161)	0.07 (0.01 to 0.14)	5653	83%	16%	0%	1%
QALY	421 (−319 to 1161)	0.01 (−0.02 to 0.04)	45,210	60%	13%	2%	25%

a bCBT: blended cognitive behavioral therapy.

b TAU: treatment as usual.

c Differences in PHQ-9 score and MINI were multiplied by −1 in order to ensure that the CE planes and cost-effectiveness acceptability curves were interpretable. Thus, differences in PHQ-9 score should be interpreted as additional points of improvement in bCBT compared with TAU and differences in MINI as cases of MDD prevented within bCBT compared with TAU.

d ΔC: The difference in costs between blended care and usual care.

e ΔE: difference in effects between bCBT and TAU.

f CER: incremental cost-effectiveness ratio.

g CE: cost-effectiveness.

h NE: northeast.

i SE: southeast.

j SW: southwest.

k NW: northwest.

l PHQ-9: Patient Health Questionnaire-9.

m MINI: Mini International Neuropsychiatric Interview.

n QALY: quality-adjusted life-years (based on EQ-5D-5L).

**Figure 4.**
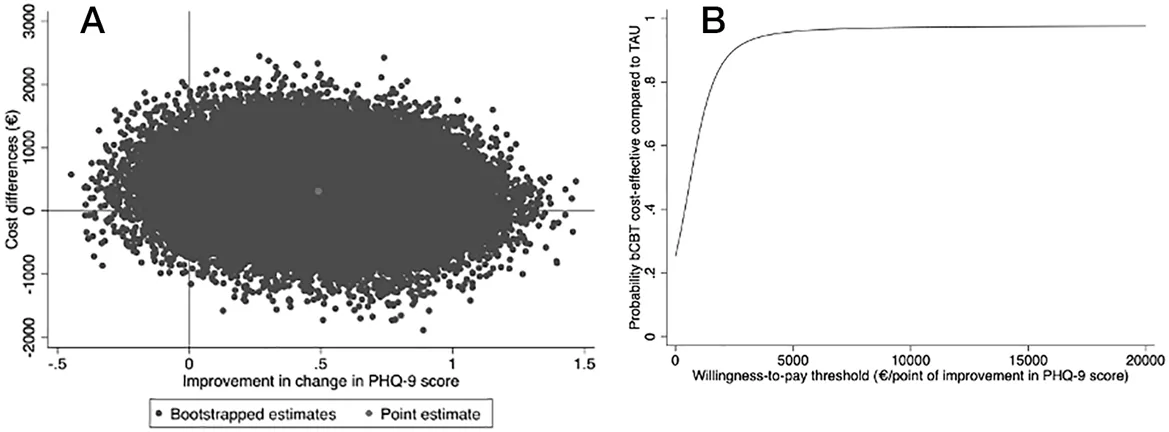
Cost-effectiveness plane of incremental costs versus change in PHQ-9 score over 12 months (A) across willingness-to-pay thresholds (B). bCBT: blended cognitive behavioral therapy; PHQ-9: Patient Health Questionnaire-9; TAU: treatment as usual.

**Figure 5.**
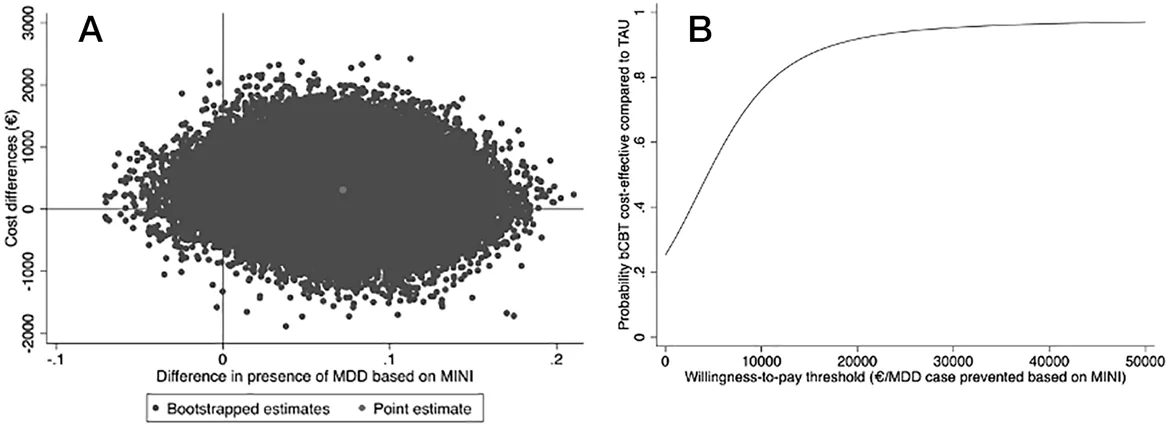
Cost-effectiveness plane of incremental costs versus change in presence of MDD (A) (MINI, 51) and across willingness-to-pay thresholds over 12 months (B). bCBT: blended cognitive behavioral therapy; MDD: major depressive disorder; MINI Mini International Neuropsychiatric Interview; TAU: treatment as usual.

**Figure 6.**
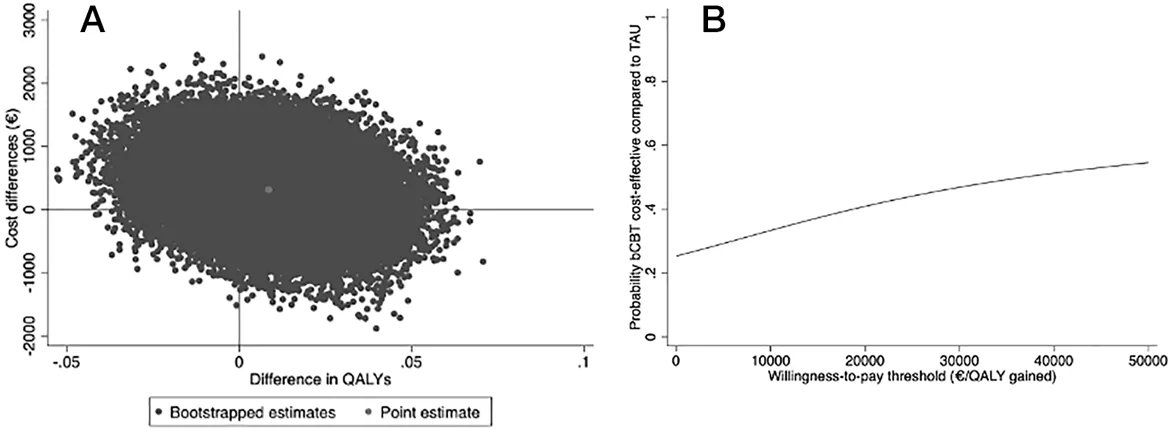
Cost-effectiveness plane of incremental costs versus change in QALYs (A) and acceptability curve across 12 months (B). bCBT: blended cognitive behavioral therapy; QALYs: quality-adjusted life-years; TAU: treatment as usual.

The cost-effectiveness plane shows that in 73% of the bootstrapped simulations, bCBT was both more effective and more expensive (Table 7: northeast quadrant of the cost-effectiveness plane). Figure 4 present the cost-effectiveness planes, plotting differences in costs against differences in effects (PHQ-9 (Figure 4A), presence of MDD (Figure 5A), and QALYs (Figure 6A) between the 2 conditions combined with the cost-effectiveness acceptability curves (Figure 4B, Figure 5B, and Figure 6B) representing the probability that bCBT is cost-effective compared with TAU across a range of WTP thresholds (WTP for PHQ-9, presence of MDD, and QALYs). As no universally accepted thresholds exist for these outcomes (except for QALYs), WTP values were varied from €0 to €50,000 to reflect a broad range of decision-making scenarios.

Cost-effectiveness planes illustrate the uncertainty around the ICER across the 4 quadrants. In Figure 4, 73% of bootstrap replications fall in the northeast quadrant, indicating that bCBT is not only more effective than TAU but also more costly (PHQ-9). It also shows that, for a 1-point improvement on the PHQ-9 and a WTP of €0, the probability of cost-effectiveness was low (0.25), rising to 0.95 at a WTP of €3,800.

A similar pattern was observed for preventing an MDD diagnosis at follow-up (12 months; Table 7). The ICER for preventing 1 MDD case was €4381, indicating that this amount would need to be invested in bCBT rather than TAU to prevent an MDD diagnosis. As shown in Figure 5, 74% of the cost-effectiveness pairs fall within the northeast quadrant (more effective and more expensive), while the remaining 26% are in the southeast quadrant (more effective and less expensive). Figure 5 also shows that the probability of cost-effectiveness ranges from 0.25 at a WTP of €0 to 0.76 at a WTP of €10,000 for every MDD episode prevented.

For QALYs, the difference between bCBT and TAU was small and not statistically significant (Table 7), with considerable uncertainty indicated by the spread of bootstrap replications across all quadrants. The ICER indicates a cost of €36,729 per QALY gained. At a €50,000 threshold, the probability that bCBT is cost-effective is 0.55 (Figure 6). In the adjusted analysis, the cost difference between bCBT and TAU increased to €421 (Table 7), but this difference was not statistically significant, and the overall results were consistent with the main analysis.

## Discussion

### Principal Findings

Our hypotheses were that bCBT for patients with MDD would be clinically noninferior to TAU and more cost-effective from a health care perspective. The results showed that while bCBT was noninferior to TAU, it was not cost-effective. The first part of our hypothesis was confirmed, as all the differential effects observed for the primary outcome (PHQ-9) in the comparison between bCBT and TAU fell within the conservative noninferiority margin (Cohen *d*=0.20). Depressive symptoms showed a significant decline up to the posttreatment assessment, and this improvement was maintained at the 12-month follow-up. The posttreatment effects for both groups also aligned well with the benchmark (*d*=1.19 [60]) for the treatment of depression with CBT in routine care settings. Additionally, the long-term effects observed in both groups are comparable with those found in meta-analyses of CBT for depression [61,62]. The bCBT group also had a lower risk of experiencing an MDD episode than the TAU group at the 12-month follow-up (OR 0.67, 95% CI 0.45-0.99). AD use was evenly distributed between the 2 groups (approximately 40%) from baseline to the 12-month follow-up, with no significant differences in proportions. Subgroup analyses indicate that participants with and without baseline AD use benefited equally from treatment. These findings are comparable with those of a meta-analysis by Cuijpers and colleagues [63], which reported a similar percentage of AD use in psychotherapeutic RCTs and found that the effects of psychotherapy for adult depression were not concurrently mediated by pharmacotherapy use. Adverse treatment effects were limited, as indicated by the reliable deterioration rates (less than 5%) for the overall group up to the 12-month follow-up. These rates compare favorably with those found in a meta-analysis of deterioration rates within psychotherapies for depression [64].

Patients’ acceptance of treatment, in terms of both the credibility and expectancy of therapy, was significantly higher for bCBT than for TAU, as was patients’ satisfaction with treatment. This may be partly explained by the higher baseline preference for the bCBT group, which, in turn, was influenced by patients’ willingness to participate in the study. An additional explanation for these higher satisfaction ratings may be that the internet-based components of bCBT better support patients’ self-management than traditional face-to-face psychotherapy, as patients are less reliant on therapists only during their treatment. This may also partly explain why, although patients and therapists valued the working alliance as strong in both conditions, patients in the bCBT group gave higher ratings than those in the TAU group. Conversely, therapists rated the therapeutic alliance significantly higher in TAU than in bCBT, except on the bond subscale. This difference may reflect therapists’ preference for face-to-face contact, including access to nonverbal cues, as well as established professional norms [65]. Nevertheless, the observed difference was relatively small, and other studies have not found such disparities [16]. Overall, these findings suggest that a strong working alliance—encompassing both human and technological components—can be established between patients and therapists in bCBT, although many participants in our study were relatively new to this mode of treatment delivery. The high alliance ratings reported by patients in both conditions are consistent with findings from the meta-analytic review by Flückiger et al [66]. For a more detailed analysis of the association between working alliance and treatment outcomes for E-COMPARED, see the study by Doukani et al [67].

Only a few RCTs have directly evaluated the clinical effectiveness of bCBT for MDD compared with active face-to-face treatment controls, limiting direct comparisons with our study. These trials recruited outpatients with MDD, compared bCBT with ftfCBT or face-to-face psychotherapy, and had broadly comparable treatment durations and intensities (12‐20 weeks). Their findings are generally consistent with ours. For example, Berger et al [68] reported that CBT supplemented with internet-based modules was superior to ftfCBT at 12 weeks posttreatment (moderate effect) [68]. Similarly, Thase et al [69] demonstrated the noninferiority of integrated bCBT compared with ftfCBT 4 months after baseline assessment, whereas Kooistra et al [23] found no significant differences in depressive symptoms between the 2 treatment conditions. Berger et al [68] and Kooistra et al [23] also reported similarly low deterioration rates, good therapeutic alliance, and high treatment acceptability, with no significant between-group differences. These findings should, however, be interpreted cautiously because the available trials were relatively small and therefore provided limited certainty regarding treatment effects.

Since completion of our study, Schaeuffele et al [70] reported the results of the large pragmatic PsyTOM trial conducted in Germany, which compared a transdiagnostic blended psychotherapy intervention with routine face-to-face psychotherapy for patients with a broad range of mental health disorders. Based on a superiority design, the authors found no evidence that blended treatment resulted in greater clinical improvement or fewer face-to-face sessions up to 6 months after randomization [70]. Although these findings differ from ours, direct comparisons should be made cautiously because the interventions and study designs differed substantially. Whereas E-COMPARED evaluated a structured, integrated bCBT protocol specifically designed for patients with MDD, PsyTOM evaluated a highly flexible transdiagnostic blended intervention delivered across multiple therapeutic orientations. In addition, PsyTOM included patients with a wide range of mental health disorders, did not restrict inclusion to formally diagnosed disorders, and allowed therapists considerable flexibility in determining both treatment duration and the use of digital components. The PsyTOM authors therefore suggested that the absence of superiority may reflect the flexibility and heterogeneity of the intervention rather than the ineffectiveness of blended treatment itself.

From a health care perspective, bCBT was associated with nonsignificantly higher health care costs than TAU over the 12-month follow-up period. Although differences in clinical outcomes were modest, they consistently favored bCBT, with slightly greater improvements in PHQ-9 scores and fewer MDD episodes. Consequently, whether bCBT can be considered cost-effective depends on how much policymakers are willing to pay for these additional health gains. Evidence on the cost-effectiveness of bCBT compared with active face-to-face treatment remains scarce. This is not unexpected, as relatively few pragmatic trials have evaluated blended care under routine clinical conditions, and economic evaluations have only occasionally been conducted alongside these trials. The few available studies have also reported inconsistent findings, with some reporting lower health care costs primarily because blended care reduced therapist time, whereas others found no significant cost differences. These inconsistencies likely reflect differences in study design, intervention characteristics, health care settings, cost perspectives, and economic evaluation methods rather than true differences in the cost-effectiveness of bCBT [23,69].

### Strengths and Limitations

This study is the first large-scale noninferiority trial to date, examining the clinical effectiveness and cost-effectiveness of bCBT compared with face-to-face TAU, predominantly ftfCBT. Noninferiority trials within routine mental health care remain scarce because of the large sample sizes required (small noninferiority margins necessitate high precision), the need for evidence-based treatment comparators, and evaluations under real-world conditions [71]. We addressed this gap by achieving a substantial sample size, using active comparators, setting a clear noninferiority margin, and conducting our RCT within routine care practices. Dropout rates in the total sample ranged from 17.5% at 3 months to 33% at the 12-month follow-up. The proportion of participants lost at 3 months (17.5%) was comparable with the average dropout reported in randomized trials of individual psychotherapy for depression in routine care [72], whereas the higher attrition at 12 months is consistent with the challenges of maintaining participant engagement during long-term follow-up. Despite this, dropout warrants careful consideration in future studies, as it may affect the validity of the findings and may indicate challenges with treatment adherence. The multifaceted nature of our study, which comprised diverse settings and technical platforms, enhances the generalizability of our results to routine care settings. Generalizability is further strengthened by including patients with and without AD use at baseline, reflecting the characteristics of patients with MDD in routine clinical practice.

Our study is not without its limitations, however. The sample size was smaller than planned due to time and budgetary constraints. However, our ability to detect noninferiority based on the predefined margin for the primary outcome during and after treatment and follow-up suggests that the sample size was sufficient to assess noninferiority [73]. This noninferiority of bCBT compared with TAU was also supported by the secondary QIDS-16 outcome at the 3- and 6-month assessments, consistent with the findings for the primary outcome (PHQ-9). However, the 2 depression measures no longer yielded consistent findings at the 12-month assessment, suggesting that the longer-term treatment effects should be interpreted with some caution. Next, all participants met the diagnostic criteria for MDD (MINI) and had a PHQ-9 score above 5 at baseline. However, because treatment was delivered within routine health care settings, participants received bCBT or TAU in either primary or specialized mental health care services. Consequently, the duration and intensity of treatment varied across settings; for example, specialized mental health care generally provided more frequent and longer treatment sessions than primary care. This between-setting heterogeneity represents a potential source of confounding that is inherent to pragmatic trials. Nevertheless, leave-one-out sensitivity analyses indicated that the overall findings were robust and were not driven by any single study site. Only the noninferiority analysis of the 12-month primary outcome showed some uncertainty after exclusion of 1 site, suggesting that the longer-term findings should be interpreted with some caution. Although this heterogeneity may have influenced the observed treatment effects, it also reflects routine clinical practice and therefore enhances the external validity of the findings.

The inability to blind patients and therapists, a common feature of psychotherapeutic studies, may also have influenced our results [74]. However, the assessors of the MINI interview were blinded, and other assessments were based on self-report. It was not possible to assess the therapists’ treatment fidelity, which may also have influenced the treatment outcomes. Additionally, some therapists delivered both treatments, which was necessary because the RCT was conducted within routine care. This may also have influenced treatment outcomes due to a preference for, or greater familiarity with, one treatment over the other [75]. Given that bCBT was still relatively new for most therapists, this could indeed have been an issue. Our cost-effectiveness analyses may also have been hampered by the sample size calculation, which was based on a noninferiority clinical margin rather than on economic outcomes. Thus, the sample size may have been underpowered to detect real effects in the cost differences between the 2 groups.

### Conclusions

To the best of our knowledge, this is the first large-scale RCT evaluating the clinical effectiveness, cost-effectiveness, and safety of bCBT for MDD in routine care, compared with TAU. The demonstration of clinical noninferiority positions bCBT as an additional acceptable treatment option for patients and therapists in routine mental health care. However, large-scale implementation will also depend on more robust evidence of its cost-effectiveness. This may improve as blended care becomes more established in practice, with increasing familiarity among patients and therapists and further optimization and standardization of delivery processes. Future studies are advised to support our outcomes. Studies are also recommended to investigate in greater depth the facilitators and contextual characteristics of bCBT for successful implementation in routine care. Future studies are also needed to explore alternative modalities of “blending,” such as face-to-face psychotherapy combined with videoconferencing, virtual reality, or AI-supported interaction between therapists and patients based on natural language models, alongside active and passive EMAs.

## Supplementary material

10.2196/72866 Multimedia Appendix 1 Recruitment and treatment settings by country.

10.2196/72866 Multimedia Appendix 2 Reliability (Cronbach α) of the questionnaires used.

10.2196/72866 Multimedia Appendix 3 Calculation of the deterioration at 3 months, 6 months, and 12 months.

10.2196/72866 Multimedia Appendix 4 Baseline sociodemographic characteristics (means, SD) per country (N=835).

10.2196/72866 Checklist 1 CONSORT 2010 checklist randomized controlled trial.

## Abbreviations

AD: antidepressant
bCBT: blended cognitive behavioral therapy
CBT: cognitive behavioral therapy
CHEERS: Consolidated Health Economic Evaluation Reporting Standards
CONSORT: Consolidated Standards of Reporting Trials
CSQ-8: Client Satisfaction Questionnaire-8
*DSM-IV*: *Diagnostic and Statistical Manual of Mental Disorders* (Fourth Edition)
E-COMPARED: European Comparative Effectiveness Research on Internet-based Depression Treatment
EE: estimated intervention effects
EMA: ecological momentary assessment
ftfCBT: face-to-face CBT
iCBT: internet-based cognitive behavioral therapy
ICER: incremental cost-effectiveness ratio
ITT: intention-to-treat
MDD: major depressive disorder
MINI: Mini International Neuropsychiatric Interview
OR: odds ratio
PHQ-9: Patient Health Questionnaire-9
QALY: quality-adjusted life years
QIDS-SR 16: Quick Inventory of Depressive Symptomatology Self-Report
RCI: Reliable Change Index
RCT: randomized controlled trial
SUS: System Usability Scale
TAU: treatment as usual
TiC-P: Trimbos/iMTA questionnaire for Costs associated with Psychiatric illness
WAI-SF: Working Alliance Inventory-Short Form
WAI-TECH-SF: Working Alliance Inventory for Online Interventions-Short Form.
WTP: willingness-to-pay
