# Supplementary material for: Clinical Effectiveness and Cost-Effectiveness of Blended Treatment for Major Depression Compared With Treatment as Usual Within Routine Care in Europe: A Noninferiority Randomized Controlled Trial
Source: Interact J Med Res. 2026 Sep 24;15:e72866. doi: 10.2196/72866 (PMC13608232; doi:10.2196/72866)
Supplement: Supplementary file 1 [file ijmr-v15-e72866-s001.pdf]

Multimedia Appendix 1

Recruitment and treatment settings by country

Country	Treatment setting	Recruitment procedure
CH	Specialized mental health care	Recruitment through two outpatient clinics (Bern and Zurich) and independent therapists' practices.
DE	Primary care	Recruitment within the waiting rooms of general practitioners.
DK	Specialized mental health care	Recruitment through the Centre for Telepsychiatry at the Mental Health Services of the Region of Southern Denmark.
ESP	Primary care & specialized mental health care	Recruitment through several primary care centers among patients who discussed their depression with their GP & outpatient clinics within hospitals.
FR	Specialized mental health care	New or regular patients recruited through CBT therapists from 11 expert centers across France.
NL	Specialized mental health care	New patients recruited through mood disorder departments of three outpatient clinics in Amsterdam and Leiden.
PL	Primary care	Recruitment through primary mental health care centers and therapists who were trained at a postgraduate CBT program conducted in five major cities (Warsaw, Sopot, Poznań, Katowice, Wrocław).
SE	Primary care	Recruitment through routine primary care clinics within Stockholm & Linköping and social media.
UK	Primary care	Recruitment through the NHS service 'Improving access to psychological therapies (IAPT)' in London, Yorkshire, Berkshire, Norfolk and Suffolk. IAPT is a service designed to increase primary mental health care by offering low-intensity treatment to GP registered adults.

Procedures

Several additional procedures were carried out, including translating the generic study protocol into national languages, monitoring progress, and developing a centralised data collection infrastructure. This also involved ensuring the completeness and quality of the data and holding periodic meetings, both in person and online, with the trial coordinators in each country to discuss outstanding issues and relevant information regarding the local context that was crucial for data management and analysis. Throughout the study, an international Scientific Advisory Commission was established.
