# Supplementary material for: Clinical Effectiveness and Cost-Effectiveness of Blended Treatment for Major Depression Compared With Treatment as Usual Within Routine Care in Europe: A Noninferiority Randomized Controlled Trial
Source: Interact J Med Res. 2026 Sep 24;15:e72866. doi: 10.2196/72866 (PMC13608232; doi:10.2196/72866)
Supplement: Supplementary file 2 [file ijmr-v15-e72866-s002.pdf]

Multimedia Appendix 2

Reliability (Cronbach's α) of the questionnaires used

	Germany	Sweden	Netherlands	UK	Spain	France	Switzerland	Poland	Denmark	Overall
**Baseline**										
PHQ-9	.69	.79	.82	.69	.64	.66	.82	.68	.66	.73
QIDS	.58	.73	.72	.84	.69	.58	.71	.54	.68	.71
CEQ_cred	.95	.75	.82	.76	.88	.55	.71	.82	.46	.86
CEQ_exp	.94	.84	.87	.90	.92	.91	.84	.92	.90	.91
AQoL	.83	NA	NA	NA	.84	.77	.82	NA	NA	.82
**3 months**										
PHQ-9	.81	.88	.87	.90	.87	.84	.87	.87	.86	.87
QIDS	.75	.82	.78	.84	.85	.79	.75	.83	.78	.81
WAI-SF	.95	.87	.93	.89	.97	.90	.94	.88	.85	.93
TAI-SF	.97	.97	.98	*N* = 0	.96	.96	.95	.94	.95	.97
CSQ	.96	.94	.91	.94	.92	.95	.76	.92	.86	.93
SUS	.81	.89	.85	.92	.81	.87	.83	.96	.72	.88
WAI-SRT	.90	.91	.83	.74	.76	.93	.86	.89	*N* = 0	.89
AQoL	.89	NA	NA	NA	.91	.89	.91	NA	NA	.90
**6 months**										
PHQ-9	.85	.89	.90	.88	.85	.87	.90	.84	.90	.88
QIDS	.80	.86	.85	.87	.84	.80	.83	.85	.81	.84
AQoL	.91	NA	NA	NA	.93	.91	.94	NA	NA	.92
**12 months**										
PHQ-9	.87	.88	.90	.86	.89	.89	.83	.88	.78	.88
QIDS	.81	.87	.82	.85	.88	.85	.69	.88	.68	.85
AQoL	.92	NA	NA	NA	.94	.91	.88	NA	NA	.93
