# Supplementary material for: Clinical Effectiveness and Cost-Effectiveness of Blended Treatment for Major Depression Compared With Treatment as Usual Within Routine Care in Europe: A Noninferiority Randomized Controlled Trial
Source: Interact J Med Res. 2026 Sep 24;15:e72866. doi: 10.2196/72866 (PMC13608232; doi:10.2196/72866)
Supplement: Supplementary file 3 [file ijmr-v15-e72866-s003.pdf]

Multimedia Appendix 3

Calculation of the deterioration at 3 months, 6 months, and 12 months

The Reliable Change Index is a psychometric norm used to determine whether the change from baseline to post-test is statistically significant. The same method was employed to calculate the Reliable Deterioration Index, which indicates whether a patient has reliably deteriorated.

$$RCI = \frac{y_{post} - y_{pre}}{\sqrt{2S_{pre}^2(1 - r_{yy})}}$$

We labeled an individual as having deteriorated if their condition worsened and if the corresponding change score passed the RCI criterion, which means that the RCI exceeded 1.96[2]. We distinguished between deterioration at 3, 6, and 12 months.

The pre-treatment standard deviations S_{pre} within the national subsamples varied from 3.97 (Denmark) to 5.62 (the Netherlands). As a result, depending on the country, individuals were labeled as having deteriorated if the increase in PHQ-9 score exceeded 2.70 (for Denmark) or 3.82 (for the Netherlands). See Table 6.1.

Table 3.1 Reliable Change Index by country

**Country**	**RCI**
Germany	2.80
Sweden	3.39
Netherlands	3.82
UK	2.98
Spain	2.94
France	3.11
Switzerland	3.31
Poland	2.96
Denmark	2.70

Both the number and percentage of patients who deteriorated were low. Within the non-imputed dataset for the entire sample, the number of patients who deteriorated was 33 (after 3 months), 28 (after 6 months), and 23 (after 12 months). The corresponding percentages were 4.8% (after 3 months), 4.6% (after 6 months), and 4.1% (after 12 months). Consequently, estimating a multi-level logistic model for the outcome of the deterioration provided unreliable estimates of the condition's odds ratios.

Table 6.2 displays the log-ratios and corresponding effect sizes estimated from generalized linear (logistic) mixed models that assessed the intervention's effect upon deterioration at 3, 6, and 12 months. As a check (sensitivity analysis), independent samples t-tests were performed upon the non-imputed data, ignoring the nesting of individuals within countries. These analyses confirmed the unreliability of the intervention effect estimates.

Table 3.2 Estimated intervention effects (top half) and effect sizes (bottom half) upon deterioration using MI data

**3 months**	**6 months**	**12 months**
Log-odds ratio		
0.372 (0.357) z=1.043 p=0.297	-0.080 (0.372) z=-0.216 p=0.829	0.474 (0.411) z=1.153 p=0.249
Effect sizes		
OR: 1.451, (0.721,2.922)<br>d: 0.206, (-0.181,0.592)	OR: 0.923, (0.445,1.913)<br>d: -0.044, (-0.447,0.358)	OR: 1.606, (0.718,3.594)<br>d: 0.262, (-0.183,0.707)

[1] Zuithoff NP, Vergouwe Y, King M, et al. The Patient Health Questionnaire-9 for detection of major depressive disorder in primary care: consequences of current thresholds in a crosssectional study. BMC Fam Pract 2010;11:98.
[2] Bauer S, Lambert MJ, Nielsen SL. Clinical significance methods: a comparison of statistical techniques. J Pers Assess 2004;82(1):60-70.
