# Supplementary material for: Clinical Effectiveness and Cost-Effectiveness of Blended Treatment for Major Depression Compared With Treatment as Usual Within Routine Care in Europe: A Noninferiority Randomized Controlled Trial
Source: Interact J Med Res. 2026 Sep 24;15:e72866. doi: 10.2196/72866 (PMC13608232; doi:10.2196/72866)
Supplement: Supplementary file 4 [file ijmr-v15-e72866-s004.pdf]

Multimedia Appendix 4

Table 4 Baseline socio-demographic characteristics (means and standard deviations) per country, total N=835

	**Germany**	**Sweden**	**Nether-lands**	**UK**	**Spain**	**France**	**Switzer-land**	**Poland**	**Denmark**	**Overall**	**bCBT**	**TAU**
**Trial Characteristics**												
Setting, N												
Primary	173	141	-	90	98	-	-	65	-	567	285	282
Specialized	-	-	84	-	-	105	50	-	29	268	138	130
N (%)	173 (20.7)	141 (16.9)	84 (10.1)	90 (10.8)	98 (11.7)	105 (12.6)	50 (6.0)	65 (7.8)	29 (3.5)	835 (100)	423 (50.7)	412 (49.3)
Randomization bCBT/TAU	86/87	73/68	45/39	45/45	48/50	51/54	26/24	33/32	16/13	423/412	-	-
**Patient characteristics**												
Age	42.94 (12.66)	34.13 (13.10)	39.96 (11.31)	33.99 (11.37)	42.06 (10.41)	44.65 (13.51)	38.66 (14.87)	35.25 (13.36)	35.17 (13.75)	39.17 (13.18)	39.52 (13.27)	38.82 (13.09)
Sex, N % female	106 (61.3)	103 (73.0)	57 (67.9)	51 (56.7)	74 (75.5)	68 (64.8)	32 (64.0)	49 (75.4)	23 (79.3)	563 (67.4)	282 (66.7)	281 (68.3)
Marital, N (%)												
Alone	66 (38.2)	61 (43.3)	35 (41.7)	58 (64.4)	43 (43.9)	56 (53.3)	20 (40.0)	23 (35.4)	12 (41.4)	374 (44.8)	199 (47.0)	175 (42.5)
Together	107 (61.8)	80 (56.7)	49 (56.7)	32 (35.6)	55 (56.1)	49 (46.7)	30 (60.0)	42 (64.6)	17 (58.6)	461 (55.2)	224 (53.0)	237 (57.5)
Education, N (%)												
Low	28 (16.2)	3 (2.1)	15 (17.9)	21 (23.3)	23 (23.5)	2 (1.9)	2 (4.0)	4 (6.2)	16 (55.2)	114 (13.7)	54 (12.8)	60 (14.6)
Middle	50 (28.9)	36 (25.5)	42 (50.0)	20 (22.2)	48 (49.0)	41 (39.0)	32 (64.0)	22 (33.8)	10 (34.5)	301 (36.0)	156 (36.9)	145 (35.2)
High	95 (54.9)	102 (72.3)	27 (32.1)	49 (54.4)	27 (27.6)	62 (59.0)	16 (32.0)	39 (60.0)	3 (10.3)	420 (50.3)	213 (50.4)	207 (50.2)
Employed, N (%)						N = 101		N = 64	N = 26	N = 827 511	N = 418	N = 409
Yes	136 (78.6)	71 (50.4)	55 (65.5)	59 (65.6)	51 (52.0)	56 (53.3)	36 (72.0)	36 (55.4)	11 (37.9)	(61.8)	248 (59.3)	263 (64.3)
No	37 (21.4)	70 (49.6)	29 (34.5)	31 (34.4)	47 (48.0)	45 (42.9)	14 (28.0)	28 (43.1)	15 (51.7)	316 (38.2)	170 (40.7)	146 (35.7)

	Germany	Sweden	Nether-lands	UK	Spain	France	Switzer-land	Poland	Denmark	Overall	bCBT	TAU
M.I.N.I., N (%)												
(MDD	173 (100)	141 (100)	84 (100)	90 (100)	98 (100)	105 (100)	50 (100)	65 (100)	29 (100)	835 (100)	423 (100)	412 (100)
Anxiety	65 (37.6)	80 (56.7)	53 (63.1)	59 (65.6)	46 (46.9)	54 (51.4)	15 (30.0)	35 (53.8)	13 (44.8)	420 (50.3)	221 (52.2)	199 (48.3)
PHQ-9	15.64 (4.14)	14.21 (5.01)	16.25 (5.65)	14.26 (4.41)	14.92 (4.35)	16.21 (4.60)	15.04 (4.92)	16.06 (4.40)	15.31 (4.04)	15.28 (4.68)	15.36 (4.82)	15.21 (4.53)
QIDS (severity)	13.95 (3.61)	14.88 (4.10)	16.05 (4.63)	11.79 (5.29)	15.92 (4.25)	15.15 (3.70)	14.18 (4.25)	16.27 (3.49)	13.28 (3.64)	14.63 (4.31)	14.62 (4.40)	14.65 (4.23)
Antidepressant medication, N (%) TiC-P*	77 (44.5)	13 (9.2)	56 (66.7)	34 (37.8)	36 (36.7)	72 (76.6)<br>N = 94	24 (48)	31 (48.4)<br>N = 64	9 (31)	52 (42.77)<br>N = 823	168 (40.29)<br>N = 417	184 (45.32)<br>N = 406
Treatment preference												
None	34 (19.7)	34 (24.1)	29 (34.5)	34 (37.8)	24 (24.5)	39 (37.1)	8 (16.0)	12 (18.0)	13 (44.8)	227 (27.2)	109 (25.8)	118 (28.6)
bCBT	133 (76.9)	66 (46.8)	39 (46.4)	45 (50.0)	69 (70.4)	22 (21.0)	40 (80.0)	51 (78.5)	6 (20.7)	471 (56.4)	241 (57.0)	230 (55.9)
TAU	6 (3.5)	41 (29.1)	16 (19.0)	11 (12.2)	5 (5.1)	44 (41.9)	2 (4.0)	2 (3.1)	10 (34.5)	137 (16.4)	73 (17.2)	64 (15.5)
CEQ_cred	15.43 (7.07)	21.97 (3.39)	18.31 (4.58)	19.49 (4.09)	16.02 (5.48)	19.73 (4.02)	20.68 (4.08)	19.55 (4.20)	20.45 (3.79)	18.32 (5.55)	19.45 (4.73)	16.97 (6.13)
CEQ_exp	14.26 (6.73)	17.37 (4.23)	16.18 (5.13)	16.80 (4.89)	17.20 (5.54)	17.56 (5.03)	17.86 (4.79)	18.21 (5.15)	18.59 (4.32)	16.60 (5.60)	17.57 (4.96)	15.45 (6.09)
UTILNL	.61 (.22)	.58 (.22)	.51 (.27)	.60 (.22)	.58 (.22)	.53 (.25)	.64 (.20)	.53 (.24)	.53 (.23)	.57 (.23)	0.58 (0.23)	0.57 (0.24)

* TiC-P: Manual Trimbos/iMTA questionnaire for costs associated with psychiatric illness.
